# Impact of Electronic Health Services on Patient Satisfaction in Primary Care: A Systematic Review

**DOI:** 10.7759/cureus.83949

**Published:** 2025-05-12

**Authors:** Tagried H Aboumoussa, Amna Hassan, Eman Ali Almarzooqi

**Affiliations:** 1 Department of Family Medicine, Dubai Health, Dubai, ARE

**Keywords:** electronic health care service, electronic health services, family medicine, patient satisfaction, primary care

## Abstract

Electronic health services (EHS) integrate telecommunications, electronic patient data, and computerized medical knowledge. The growing implementation of EHS in primary care underscores the necessity to comprehend its effect on patient satisfaction and highlight areas for improvement. This systematic review study aims to evaluate the impact of EHS on patient experiences in a primary care setting. This review was conducted in accordance with the Preferred Reporting Items for Systematic Reviews and Meta-Analyses (PRISMA) guidelines. A series of searches was conducted until November 2024 in the following databases: PubMed, Science Direct, and the Cochrane Library. Two independent reviewers extracted the data from eligible studies using a standardized extraction sheet. The inclusion criteria encompassed randomized controlled trials (RCTs), observational studies involving adult patients who employed EHS interventions, including electronic health records (EHRs), telemedicine, patient portals, or online appointment systems. The Cochrane Risk-of-Bias tool and Newcastle-Ottawa Scale have been used to assess the risk of bias of included studies. Ten studies involving participants ranging from 52 to 203,903 were included. It was seen that increased provider focus on EHR use, including prolonged silence and gaze at the screen, negatively influenced patient-centered communication and involvement. Nonetheless, in a variety of contexts, the use of EMRs enhanced patient satisfaction with clinical consultations, services, and overall healthcare experiences. Effective prescription and referral procedures, improved communication, and reduced wait times were among the improvements. Patient portals and EHS demonstrated increased satisfaction with healthcare quality, particularly among patients with long-term provider relationships. Socioeconomic factors, such as age, education, and income, influenced preferences for communication modes like portals, phone calls, and text messages. This systematic review demonstrates the transformative potential of EHS in enhancing patient satisfaction within primary care. EHR/electronic medical record (EMR) systems were associated with better service efficiency and patient satisfaction, despite challenges in balancing provider interaction with technology use. By improving access, communication, and efficiency, EHS can play a pivotal role in advancing patient-centered care. However, challenges related to provider communication, interoperability, and health equity highlight the need for thoughtful implementation and continuous refinement of these tools.

## Introduction and background

The integration of technology into healthcare has transformed the healthcare delivery systems, with electronic health services (EHS) becoming a major component in the last decade, and their adoption by healthcare professionals is rising. EHS offers computerized medical knowledge, electronic patient information, and telecommunications to enhance the quality, safety, and efficiency of healthcare delivery [1]. This generic category encompasses electronic health records (EHRs) such as digital versions of patients' medical charts, telemedicine, patient portals, and online appointment booking systems, all of which have the potential to make healthcare more organized and available, which leads to more safety, effectiveness, and equitability and may be a significant step in enhancing both the quality and efficacy of health care services [1].

EHRs are software programs developed to centralize and simplify patient data storage, retrieval, and utilization. These systems have been recognized for lowering healthcare costs, increasing patient safety, and enhancing the quality of care. EHRs minimize clinicians' workloads by combining patient information, reducing medical errors, and supporting evidence-based clinical decision-making procedures [2-5]. For instance, EHRs can provide alerts and reminders for preventive care and screenings; hence, it is easier for healthcare practitioners to follow best practices and guidelines. Moreover, EHRs enable real-time monitoring of patient data, allowing for early identification of potential health threats and timely interventions [6]. These advantages highlight the critical role of EHRs in contemporary healthcare systems. Additionally, the transition from paper-based medical records to EHRs has been associated with numerous benefits, but patient satisfaction with these systems remains a significant area of inquiry, highlighting the need to balance technological integration with user experience [7].

Another key component of EHS is telemedicine, which involves delivering medical care and consultation services remotely through telecommunications technologies. Using health apps for scheduled follow-up visits makes medical practitioners and patients more effective, which also increases the likelihood of follow-up, minimizes missed appointments, and improves patient outcomes [8]. Telemedicine has gained popularity as a beneficial alternative to direct consultations; primarily, it can eliminate geographical barriers, and it has become a viable option to deliver care to remote and rural areas. The COVID-19 pandemic-related closure of in-person healthcare services resulted in the usage of telehealth services as a necessity rather than a choice [8,9]. The transition to remote care has also raised questions about the extent to which telemedicine satisfies patients' expectations and requirements.

Studies conducted in different countries pointed to the impact of EHS on the healthcare system. While EHS offers undeniable advantages, its implementation has also introduced challenges, particularly with respect to patient satisfaction. Several studies have explored the effects of EHS adoption on patients’ experiences in primary care. Some studies discovered a significant drop in patient satisfaction domain levels, particularly in areas pertaining to accessibility, like making appointments and contacting clinics [10].

In many developing countries, the primary healthcare center, which offers primary healthcare services to a large number of patients, is an essential part of the healthcare system. The growing use of EHS in primary care emphasizes the necessity of comprehending how they affect patient satisfaction and pinpointing areas in need of development. Despite the potential benefits of EHS, there is limited research on its impact on patient satisfaction in primary healthcare settings. This study aims to fill this gap in the literature by investigating the impact of EHS on patient satisfaction in primary healthcare centers by focusing on how different components of EHS, including EHRs, telemedicine, patient portals, and online scheduling systems, influence patient experiences in primary care settings.

## Review

Methods

Inclusion Criteria and Exclusion Strategy

The inclusion criteria encompassed randomized controlled trials (RCTs) and observational studies involving adult patients who employed EHS interventions, including EMRs, telemedicine, patient portals, or online appointment systems. Studies reporting on patient satisfaction outcomes, including access, communication, workflow, and overall satisfaction, were included. Observational studies (cohort and cross-sectional) and RCTs published in the English language during 2014-2024 have been included. We excluded studies conducted in non-primary care settings or studies that included non-EHS interventions. We also excluded non-English-language studies, non-original (previously published) articles, abstracts, conference papers, short communications, reviews, and case reports.

Search Strategy

The review was conducted in adherence with the Preferred Reporting Items for Systematic Reviews and Meta-Analyses (PRISMA) guidelines in all steps. We searched electronic databases such as PubMed, Cochrane Library, and Science Direct for studies published between January 2014 to November 2024. The search strategy was as follows: "patient portals" OR "electronic health services" OR "remote patient monitoring" OR "electronic medical record" OR "paper medical record" OR "medical records systems" OR "mHealth" OR "telemedicine" OR "electronic" OR "SMS" OR "Chatbot" AND "patient experience" OR "patient satisfaction" OR "patient engagement" OR "physician-patient communication" OR "health care quality" OR "health care outcomes" AND "primary healthcare" OR "primary care" OR "family practice", and we used filters that limited the results to English-language studies published in 2014-2024. Additionally, reference lists of relevant articles were reviewed to ensure comprehensive coverage of the literature.

Screening and Data Extraction

Articles retrieved through the search strategy underwent a multi-step screening process. First, irrelevant titles or abstracts were excluded during the title and abstract screening phase. Second, the full texts of potentially relevant articles were reviewed to determine their compliance with the inclusion criteria. Titles and abstracts were organized and scrutinized for duplicates using reference management software (EndNote X8; Clarivate Plc, London, United Kingdom) [11]. A dual screening approach was employed, with one reviewer screening titles and abstracts and another conducting comprehensive examinations of full texts. Disagreements were resolved through discussion.

The data extraction process was carried out by two authors independently. Any disagreement was resolved with a third author. Data were collected with the spreadsheet software Microsoft Excel version 2412 (Microsoft Corporation, Redmond, Washington, United States). Baseline characteristics of the included studies, such as author, publication year, country, study design, participant demographics such as age, gender, and type of EHS implemented, and outcome measures such as patient satisfaction scores, ease of access, communication effectiveness, and workflow improvements, were extracted from the selected studies.

Quality Assessment

Two authors independently assessed the methodological quality of the included studies. Any disagreement was resolved with a third author. To evaluate the risk of bias in the included RCT studies, we employed the Cochrane risk-of-bias tool [12]. In seven domains, including random sequence generation, allocation concealment, participant and staff blinding, outcome assessment blinding, incomplete outcome data, selective reporting, and other potential biases, bias was rated as "high risk," "low risk," or "unclear".

The quality score of observational studies was assessed using the Newcastle-Ottawa Scale (NOS) [13]. The NOS tool is a standardized tool designed to assess the quality of non-randomized observational studies, particularly cohort and case-control studies, in systematic reviews and meta-analyses. It evaluates studies across three broad domains: Selection, Comparability, and Outcome (for cohort studies) or Exposure (for case-control studies). The Selection domain (up to 4 stars) assesses the representativeness and selection of study groups, ascertainment of exposure, and whether the outcome of interest was present at the start of the study. The Comparability domain (up to 2 stars) evaluates the extent to which the study design or analysis controlled for confounding variables, awarding one star for controlling the most important factor and another for additional factors. The Outcome or Exposure domain (up to 3 stars) examines the adequacy and objectivity of outcome or exposure assessment, the duration and adequacy of follow-up (for cohort studies), or the response rate and ascertainment methods (for case-control studies). Each study can receive a maximum of 9 stars, and studies scoring 7 or more stars are typically considered high quality. The NOS provides a transparent and reproducible framework for assessing study bias and methodological rigor in observational research.

Results

Search Results

**Figure 1:**
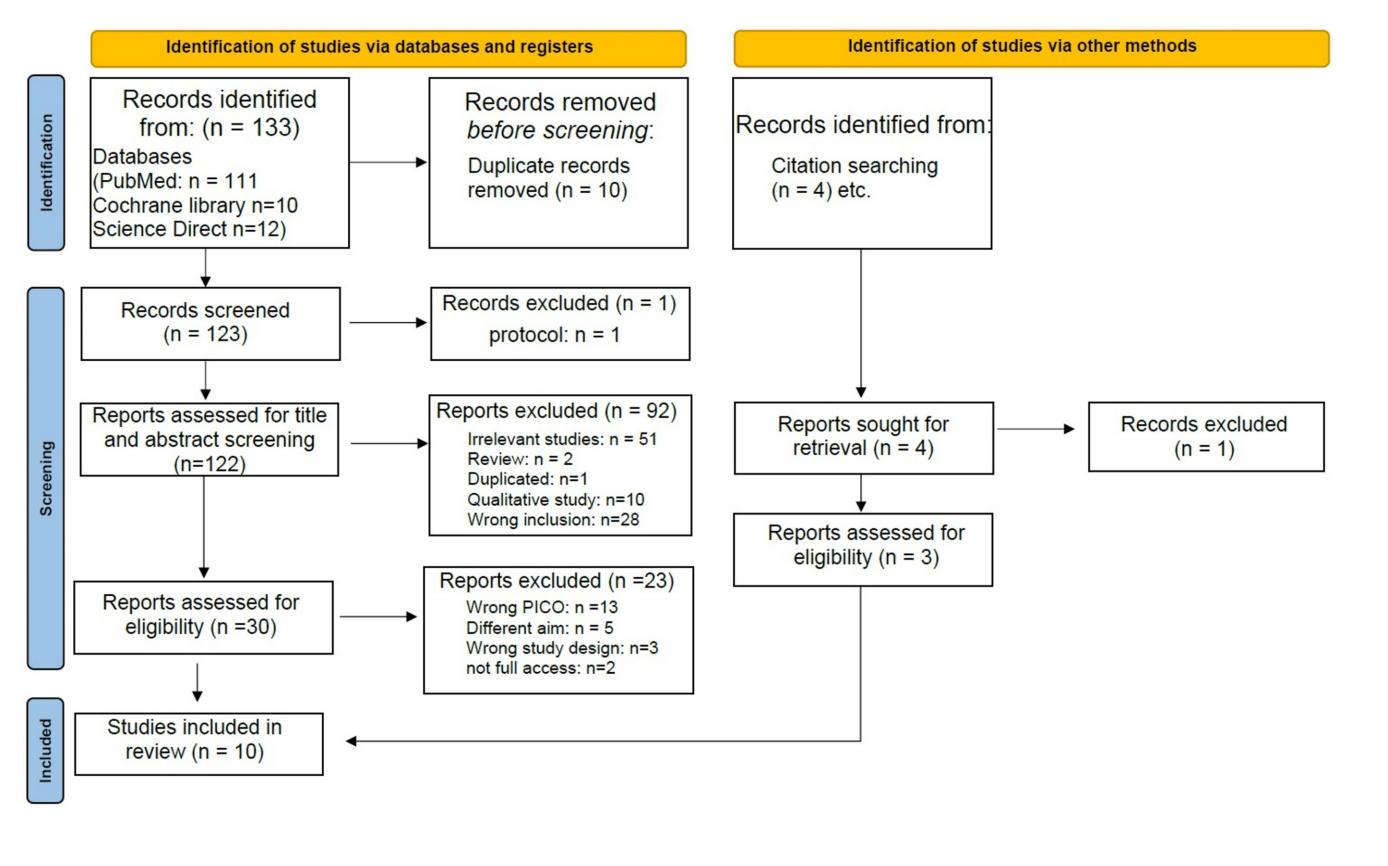
PRISMA flow diagram of the study selection process PRISMA: Preferred Reporting Items for Systematic Reviews and Meta-Analyses

The search strategy yielded 133 citations (111 from PubMed, 10 from Cochrane Library, 12 from Science Direct), of which 10 were removed as duplicates. After titles and abstracts screening, 30 studies met the eligibility criteria, and others were excluded for not fulfilling the inclusion criteria. After the final full study screening, a total of 10 publications with available full texts remained [14-23]. The PRISMA flow diagram of the study selection process is illustrated in Figure 1.

Results of Quality Assessment

The NOS was used to assess the quality of observational studies, with Legler et al. [19] achieving the highest score of 8. Other studies received "Satisfactory" ratings, while Vitale et al. [18] received an "Unsatisfactory" rating. The Cochrane Risk of Bias tool showed a "High" overall risk of bias, affecting the reliability of the studies (Tables 1-2).

**Table 1:** Cochrane risk of bias for included randomized studies

Author	Year	Random sequence generation	Allocation concealment	Blinding of participants and personnel	Blinding of outcome assessment	Incomplete outcome data	Selective reporting	Other bias	Overall
Rollman et al. [14]	2017	Low	Low	High	Low	Low	Low	High	High
Shimada et al. [15]	2020	Low	Unclear	High	High	Low	Low	High	High

**Table 2:** Quality assessment of Newcastle–Ottawa scale (NOS) for included observational studies

Author	Year	Selection					Comparability	Outcome			Quality
		Representativeness of the Sample	Sample size	Non- Respondents	Ascertainment of the Exposure	The Subjects in Different Outcome Groups are Comparable		Assessment of outcome	Statistical analysis	Total score	
Street et al. [16]	2014	1	0	0	1	1		1	1	5	Satisfactory
Al-Dahshan et al. [17]	2017	1	1	1	1	0		1	1	6	Satisfactory
Vitale et al. [18]	2019	1	1	0	1	0		0	1	4	Unsatisfactory
Legler et al. [19]	2019	1	1	1	2	1		1	1	8	Good
Wali et al. [20]	2020	1	1	0	1	0		1	1	5	Satisfactory
Cross et al. [21]	2021	1	1	1	1	1		0	1	6	Satisfactory
Fridman et al. [22]	2023	1	1	0	1	1		1	1	6	Satisfactory
Asiri et al. [23]	2024	1	1	0	1	0		1	1	5	Satisfactory

Baseline Characteristics of Included Studies

The major baseline characteristics of the included studies are shown in Table 3. The publication year of the eligible studies ranged between 2014 and 2024. The number of patients ranged from 52 to 203,903, reflecting considerable variability in scale. Study designs varied, encompassing RCTs, retrospective observational, cross-sectional, and exploratory surveys. The studies were conducted in the United States, Saudi Arabia, and Qatar. The mean age of participants was predominantly in the 60s, aligning with the typical demographic of primary care patients, and most studies showed a male predominance, with gender ratios ranging from 53% male in Asiri et al. [23] to 96.8% male in Street et al. [16].

**Table 3:** Baseline characteristics of included studies *Reported age values are presented as mean ± standard deviation in accordance with common reporting practices across studies; in some cases, age is also expressed as a distribution

Author	Country	Study publication year	Study design	Study period	Total participants	Age of total participants (years)*	Gender (M/F), %
Street et al. [16]	United States	2014	-	-	125	60.4±13.4	96.8%/3.2%
Al-Dahshan et al. [17]	Qatar	2017	Descriptive cross-sectional	2015-2016	52	<20: 4 (7.7%); 20-29: 6 (11.5%); 30-39: 28 (53.8%); 40-49: 10 (19.2%); 50 and over: 4 (7.7%)	53.8%/46.2%
Rollman et al. [14]	United States	2017	Randomized controlled trial	2005-2007	cases: 250	44.6±11.2	26%/74%
					control: 79	42.1±11.1	38%/62%
Legler et al. [19]	United States	2019	Retrospective cross-sectional	-	203,903		94.8%/5.2%
Vitale et al. [18]	United States	2019	Exploratory survey	2016-2017	pre: 200 post: 215	-	-
Shimada et al. [15]	United States	2020	Randomized controlled trial	2016-2018	cases: 595	<50: 20.8%	90.3%/9.7%
					control: 600	<50: 22%	89.8%/10.2%
Wali et al. [20]	Saudi Arabia	2020	Cross-sectional	July 10 to December 31, 2018.	377	35.76±11.56	35%/65%
Cross et al. [21]	United States	2021	Cross-sectional	2019	158	71.4	33.5%/66.5%
Fridman et al. [22]	United States	2023	Cross-sectional	2019	133	64.19±7.87	37%/63%
Asiri et al. [23]	Saudi Arabia	2024	Cross-sectional	2022	249	18: 91 (36.5%); 30: 85 (34.1%); 40-63: 73 (29.3%)	53%/47%

Patient Satisfaction Outcomes

Patient satisfaction outcomes based on the data derived from 10 studies following the implementation of EHS in primary care are presented in Table 4. Regarding patient-centered communication, reduced gaze time and extended silences had a negative impact on patient engagement (silence time negatively associated with communication effectiveness: coefficients = −27.7, SE = 4.58, p < 0.0001), according to Street et al. [16], who found that increased provider use of EHRs had a negative impact on patient-centered communication. The effectiveness of primary care physicians' communication declined with increased EHR gaze time and prolonged quiet.

**Table 4:** Patient satisfaction outcomes following implementation of EHS in primary care PCP: Primary Care Physician; EHR: Electronic Health Record; EMR: Electronic Medical Record; CC: Clinical Care; PS: Patient Satisfaction; I: Intervention; SM: secure messaging; SAP: Supported Adoption Program; PPR: patient-provider relationship; HRQoL: Health-Related Quality of Life; ES: Effect Size; PHC: Primary Healthcare; PMR: Paper Medical Records; OS: Overall Score; TS: Total Score; HS: Health Status; SDOH: Social Determinants of Health; EHS: Electronic Health System

Author	Aim	Healthcare service system	Patient satisfaction score	Method	Other findings	Conclusion
Street et al., [16]	The study examined the effects of PCPs' nonverbal interaction with the EHR including looking at the computer, rate of mouse click/scrolling activity, and silence during computer use - on observer ratings of the providers' patient-centered communication, patient involvement in the consultation, and provider control of conversation.	EHR	PS :4.64 ±0.39 CC: 49.4 ±7.90 I: 27.7 ±5.88	Multivariable analysis	Patient center communication: Gaze time at EHR (%): - (Coefficients=−17.1; SE: 3.60, p<0.0001). Visit length: - (coefficients= 0.003, SE= 0.001, p=0.0003). Silence time (percentage): -(coefficients =−27.7, SE=4.58, p<0.0001). Patient involvement: - Visit length:-( coefficients=0.002, SE=0.001, p=0.002). Silence time (%) :-(coefficients=− 16.4, SE= 3.83, p<0.0001). Conversational control (provider talk time over patient/companion talk time) Silence time (%) :(coefficients=2.46, SE= 0.94, p=0.010).	PCPs communication effectiveness decreases when they spend more time focused on the computer and when consultations involved prolonged periods of silence.
Al-Dahshan et al., [17]	The study assessed the level of patient satisfaction regarding PHC services after the implementation of an EMR system.	EMR	High	Likert scale and percentages	time spent at the registration desk (76.9%), before seeing a physician (65.4%), physician used the computer (76.9%), physical examination (69.3%|), laboratory investigation (73.1%), collecting medication at the pharmacy (65.4%)	Overall patient satisfaction was relatively high.
Rollman et al., [14]	The study examined the impact and 12-month durability of a centralized, telephone-delivered, stepped CC intervention for treating anxiety disorders across a network of PCP.	EMR	Increases	Intent-to-treat analyses	HRQoL [ES]: 0.38 [95 % CI: 0.13–0.63]; p= 0.003), anxiety (ES: 0.30, 95 % CI [0.05–0.55]; p= 0.02), and mood (ES: 0.45, 95 % CI [0.19–0.71] *p* = 0.001).	EHS led to improvements in patient satisfaction, (mental HRQoL, anxiety and mood symptoms).
Legler et al., [19]	The study assessed the relationship between the PC provider being an early adopter of JLV and patient perception of the provider’s knowledge of their medical history.	EHR	Increases	Multivariate logistic regression	Provider has previously used JLV: (adj OR: 1.14, 95%CI:1.08-1.21;* p* < 0.000). Length of PPR: 60+ months (adj OR:2.66;95%CI :2.58–2.75) PA/NP (adj OR: 1.09; 95%CI:1.06–1.13). PPR and provider used JLV for 3 + years (adj OR 1.23, *p* < 0.000). PPR and provider used JLV for < 3 years (adj OR :1.11; 95%CI 1.06–1.13; p < 0.05).	Patient longer term relationships, is associated with increased patient satisfaction and greater patient perceptions of provider knowledge.
Vitale et al., [18]	To improve the telephone communication experience for patients in a PCP.	EMR	Increases	Student’s t-test or chi-square test.	•questions answered (79.9% often or always after vs. 67.4% prior, p<0.01). •treated with respect (94.8% often or always after vs 89.4% prior, p=0.01). •easier to make an appointment (75.8% often or always after vs 69% prior, p=0.03). •reporting urgent care or emergency department visits (33.8% before vs 31.9% after, p=0.68). •Leaves message when calling (42.3% often or always after vs 42.4% prior, p=0.70). •Receives reply within 24 hours (38.6% often or always after vs 44% prior, p=0.13).	Several improvements in the patient experience
Shimada et al., [15]	The study examined differences in SM adoption rates between SAP recipients and controls at 9 months and 21 months and to assess perceived provider autonomy support and self-report of telephone communication with clinical teams.	SM/SAP	NR	Chi-square tests, t test, multivariable regression analysis,	Adoption of Secure Messaging at 9 Months: (17.0% vs 6.7%; *p*<.001). At 21 months: (23.7% vs 13.5%; *p<*.001). Health care climate scale:( mean difference: 0.3; 95%CI: (0.1 to 0.5), p= .006). communicate phone autonomy supportive: (68.8% vs 76.0, difference: –7.2,95%CI: (–14.5 to 0.0), p=.007). Easy to communicate :(68.8% vs 76.0%, difference: 6.2; 95%CI: (–1.7 to 14.1), p=.05).	SAP was successful in increasing use, significant effect on SM adoption and improved perceptions of provider autonomy support.
Wali et al., [20]	Study explored patient satisfaction with the EMR compared to the PMR of patients attending 5 PHC in the Western Region of Saudi Arabia.	EMR/PMR	OS: 3.708	Chi-Square test, Likert scale of 1 to 5.	PS with the Medical Consultation (before vs after EMR): - Physician attention :77%/82.3%; p	Significant improvement in patient satisfaction during the clinical consultation and overall satisfaction with various PHC services.
Cross et al., [21]	Study analysed whether older adults’ assessment of PC quality differs across levels of patient portal engagement and whether perceptions of how well their provider uses the EHR to support care moderates this relationship.	EHR	NR	Multivariate ordinary least squares regression	Quality of care: - High EHR value perception: [coefficient (SE): 0.288 (0.074), p<0.001]. Length of relationship with primary care physician [10+ years: [coefficient (SE)= 0.350 (0.086); P< 0.001].	Moderate portal use among older adults is associated with lower satisfaction with care, and the most sensitive to perceptions of how effectively providers use the EHR to enhance patient provider relationship.
Fridman et al., [22]	study explored stated preferences by communication modes through the lens of SDOH to gauge acceptability and equity implications for future interventions.	patient portal (71%), email (47%), telephone calls (41%) and SMS text messages (30%).	NR	Pearson chi-square	age (younger vs. older): -(82% vs. 63%; *p*=0.02), gender: - (58% vs 38%, * p*=0.04), race (black vs white respondents):- (60% vs 42%; * p*=0.046); higher education(college degree or more):-(85% vs 61%; p=0.048), income(higher vs lower):-(89% vs 60%; * p*<0.01) are influencing patients preferences for receiving healthcare information via different EHS.	To optimize health communication and reach a socioeconomically diverse population, telephone calls should be added to electronic communication, especially for people with less income and education.
Asiri et al., [23]	Study evaluated the effect of EHS on patient satisfaction at the PHC in Abha, southwestern Saudi Arabia.	EHR	Gender (OS): -Female:1±0.6 Male: 0.7±0.8, p= 0.027, HS (p = 0.015), TS (p = 0.036). Educational level (OS): - Secondary school: 1.2±0.6, Undergraduate: 0.8±0.7, Diploma: 1±0.6, Postgraduate: 0.6±0.9, p= 0.003, TS (p =0.032), HS (p = 0.001). Educational background (HS): - healthcare sciences: 0.7±0.8, Other educational fields: 0.9±0.8, p= 0.028.	Chi-square tests and logistic regression.	86.3% of participants used EHS for appointments (Sehhaty);73.5% as well. 71.5% of participants agree satisfaction with the care they received during their visit.	The study results suggested that the implementation of EHS in PHCCs in the southwestern region of Saudi Arabia has the potential to enhance patient satisfaction.

Regarding patient satisfaction with EMR, Al-Dahshan et al. reported high satisfaction levels, with over 70% of participants expressing satisfaction with a variety of care-related factors, such as laboratory tests and registration efficiency [17]. Likewise, Wali et al. determined that the deployment of EMR significantly increased satisfaction measures such as clinical encounter time (73.8% to 80.4%, p < 0.001) and physician attention (77% to 82.3%, p < 0.001) [20].

Regarding healthcare quality and patient satisfaction, Rollman et al. discovered that the use of a telephone-delivered, stepped care model integrated with EHR resulted in substantial gains in patient-reported mental health outcomes, mental health-related quality of life, anxiety symptoms, and mood (e.g., health-related quality of life (HRQoL) effect size = 0.38, 95%CI: 0.13-0.63, p = 0.003) [14]. Vitale et al. observed improvements in telephone contact experiences, including increased patient satisfaction with respect (94.8% vs. 89.4%, p = 0.01) and feeling their questions were answered (79.9% vs. 67.4%, p < 0.01) [18].

On the other hand, provider relationships and EHR usage were reported in one study. Legler et al. found that patients with longer-term provider relationships and those whose providers used integrated viewers for over three years reported higher satisfaction scores [19]. Providers who were early adopters of EHR systems were perceived as more knowledgeable about patient medical history (adjusted OR = 1.14, 95%CI: 1.08-1.21, p < 0.000). Meanwhile, Cross et al. studied the relation between older adults and patient portals and found that elderly persons who used portals somewhat were less satisfied with their care [21]. However, the association between portal use and care quality was favorably mediated by providers' evaluations of effective EHR use (coefficient = 0.288, SE = 0.074, p < 0.001). Furthermore, sociodemographic influences, including age, gender, race, income, and education, and their impact on communication preferences were explored by Fridman et al. [22]. In order to maximize health communication for people with lower incomes or educational levels, phone calls were advised.

Lastly, regional EHS implementation was also of importance. According to Asiri et al., the majority of participants expressed satisfaction with appointments (86.3%), ease of usage, and overall care during visits (71.5% agreement), and 86.3% of individuals used EHS for appointments [23]. Satisfaction was highly influenced by gender and educational attainment, with women and those with a secondary education reporting higher levels of satisfaction.

Overall, the reviewed studies highlight the potential of EHS to enhance patient satisfaction in primary care settings. The majority of studies documented notable increases in access, efficiency, and patient involvement after EHS deployment, despite certain difficulties, such as detrimental effects on communication during EHR use.

Discussion

Evidence regarding the effect of EHS on patient satisfaction in primary care settings is compiled in this systematic review. Secure messaging, online appointment scheduling, patient portals, electronic medical records, and other EHS interventions are revolutionary instruments for enhancing patient-centered care.

In line with the current findings showing the importance of EHS in the improvement of patient satisfaction, a previous systematic review suggested a positive association between patient access to EHRs and healthcare engagement [24]. The implications of these findings for healthcare providers, policy makers, and patients should be considered, highlighting the potential benefits and challenges associated with implementing and promoting patient access to EHRs.

The findings of this systematic review demonstrate that EHS play a significant role in shaping patient satisfaction in primary care. Applying Donabedian’s framework of Structure-Process-Outcome, we can more clearly understand the mechanisms through which EHS affects patient experiences and perceptions [25].

Structure: Technological Infrastructure and Accessibility

The structural elements of care refer to the settings, tools, and resources available to support healthcare delivery. EHS interventions such as EMRs, patient portals, secure messaging platforms, and digital applications like Sehhaty (Ministry of Health (Saudi Arabia), Riyadh, Saudi Arabia) represent a transformative shift in the structural foundations of primary care. Patients reported higher satisfaction with facilities that transitioned from paper-based to electronic systems, as shown by studies by Wali et al. [20] and Al-Dahshan et al. [17]. However, disparities in access, especially among older adults, low-income groups, and individuals with limited digital literacy, suggest that structural inequities persist. As Fridman et al. [22] and Asiri et al. [23] indicate, factors such as education level, income, and gender affect both satisfaction and adoption, raising equity concerns that must be addressed through inclusive EHS design and implementation.

Process: Communication, Engagement, and Workflow

Process indicators reflect the interactions between patients and providers, as well as the mechanisms of care delivery. In this review, EHS was found to enhance communication and efficiency, two key contributors to patient satisfaction. Tools like secure messaging and patient portals empowered patients, aligning with principles of autonomy and shared decision-making [15]. However, EHS can also disrupt provider-patient communication. As noted by Street et al., excessive focus on screens during consultations undermines engagement and reduces the quality of interpersonal interaction [16]. Moreover, issues such as complex user interfaces or provider distraction during data entry detract from the patient experience, despite technological gains. These findings emphasize that process improvements must balance efficiency with relational care, underscoring the need for user-centered design, ambient documentation technologies, and training for providers to maintain patient engagement during EHS use.

Outcomes: Patient Satisfaction and Perceived Quality of Care

Outcomes in Donabedian’s model pertain to the end results of healthcare processes, including patient satisfaction. The review consistently showed that EHS improves satisfaction through better access, transparency, and engagement with care. Al-Dahshan et al. [17] and Asiri et al. [23] reported high satisfaction scores linked to EHS-facilitated services such as streamlined registration, efficient consultations, and laboratory testing.

Nonetheless, satisfaction was not universal. Certain service areas, such as health education, lag behind, suggesting that EHS must be integrated into a broader framework of quality improvement. Furthermore, perceived satisfaction varied among different patient groups, revealing that outcome measures must be stratified by sociodemographic characteristics to ensure that care enhancements are equitable and impactful.

Enhanced Patient Satisfaction and Workflow Efficiency

The majority of the studies in this systematic review show that EHS significantly improves patient satisfaction. Implementing tools such as EMRs and secure messaging systems improved patients' perceptions of care quality, access, and communication with healthcare providers. For instance, Wali et al. [20] and Al-Dahshan et al. [17] demonstrated higher patient satisfaction scores in primary care centers that transitioned from paper medical records to EHRs. Al-Dahshan et al. specifically reported high levels of satisfaction with key service areas, including registration efficiency, physician consultations, and laboratory testing [17]. However, less than two-thirds of patients expressed satisfaction with the health education provided by physicians, highlighting the need for targeted improvements in specific areas of care. This study emphasizes patients' general acceptance of EMR systems while identifying actionable areas for enhancing their overall experiences. 

Similarly, Asiri et al. examined patient satisfaction with EHS in southwestern Saudi Arabia, particularly with digital applications like Sehhaty [23]. Their study found that 71.5% of participants were satisfied with care facilitated by EHS, with female participants reporting higher satisfaction levels than their male counterparts. Interestingly, individuals with higher educational attainment expressed lower satisfaction compared to those with less formal education, suggesting that perceptions of EHS may vary based on educational background. These findings indicate the importance of tailoring EHS implementations to meet the diverse expectations and needs of different patient groups.

While most studies report higher satisfaction with EHS among individuals with greater educational attainment due to increased digital literacy and engagement [26,27], the findings in this review diverge, showing that patients with lower education levels reported higher satisfaction. This discrepancy may be attributed to several factors. Higher-educated individuals often have elevated expectations for system performance, usability, and responsiveness, making them more critical of limitations in EHS platforms. They may also possess greater awareness of system shortcomings, such as interoperability issues or data privacy concerns. Conversely, patients with less formal education may perceive greater relative benefits from even basic EHS features like streamlined appointments and access to test results. These findings underscore the need to tailor EHS design and communication strategies to the diverse expectations and digital experiences of different patient groups.

Additionally, tools like patient portals and secure messaging, as highlighted in Shimada et al. [15], empower patients by giving them greater control over their health management. These findings align with the principles of shared decision-making and autonomy, which are increasingly recognized as critical to patient satisfaction and engagement. Enhanced autonomy in health management, facilitated by EHS, reflects broader trends in digital health innovation and supports the growing emphasis on patient-centered care.

Challenges to Communication and Provider-Patient Interaction

While EHS enhances satisfaction in many domains, it also introduces complexities, particularly in communication. Studies such as that by Street et al. reveal how prolonged provider engagement with EHRs during consultations can detract from patient-centered communication, leading to decreased patient involvement and engagement [16]. Providers who spent more time gazing at computer screens or introduced long periods of conversational silence received lower ratings for communication and engagement. This reflects broader challenges in maintaining the relational aspects of care when technology becomes a prominent feature of the consultation room. Successful communication, essential for building trust and comprehension between healthcare providers and patients, may be inadvertently compromised when providers are overly dependent on or become distracted by EHS tools while interacting with patients.

The interoperability of EHR systems plays a critical role in addressing some of these difficulties by enhancing workflow efficiency and minimizing redundancies. Legler et al. examined the use of the Joint Legacy Viewer (JLV), an integrated system for viewing EHRs across healthcare systems, and found that providers who utilized JLV were perceived as more knowledgeable about their patients’ medical histories [19]. This effect was particularly pronounced in long-term patient-provider relationships, highlighting the potential of interoperable systems to enhance care quality and improve patient experiences by facilitating seamless information exchange. However, interoperability alone cannot resolve the issue of provider distraction during consultations, emphasizing the need for user-friendly designs that integrate smoothly into clinical workflows.

In addition to interpersonal communication challenges, EHS implementation may also affect the dynamics of shared decision-making. Patients may feel less engaged in consultations when providers are preoccupied with navigating complex systems or inputting data into EHRs [24,26]. This aligns with evidence suggesting that patients often perceive a loss of personal connection when technology becomes a central focus of the clinical encounter. Balancing operational efficiency with relational care requires not only technological improvements but also behavioral adjustments by providers to ensure patients feel seen, heard, and valued during their visits [27].

Technological innovations also hold promises for alleviating communication challenges. Integrating voice-activated or ambient documentation tools could reduce the cognitive and time burden on providers, allowing them to focus more on the patient. Such tools have been shown to streamline documentation processes, freeing up provider attention for relational aspects of care. Moreover, optimizing EHR user interfaces to minimize navigation complexity can further enhance provider efficiency while maintaining communication quality [28,29].

Finally, it is essential to consider how EHS tools are designed to facilitate, not hinder, relationship-building. Human factors engineering can play a pivotal role in creating systems that prioritize intuitive interactions and reduce friction in clinical workflows. For example, smart notifications that alert providers to critical patient needs without overwhelming them with non-essential data can help maintain focus during consultations. Additionally, involving both providers and patients in the design and testing of EHS tools can ensure that these systems support rather than detract from patient-centered care [30,31].

Equity and Disparities in EHS Adoption

The adoption of EHS has the potential to bridge gaps in healthcare access and improve outcomes for diverse patient populations. However, this review highlights significant disparities in EHS utilization based on sociodemographic characteristics, which could inadvertently exacerbate existing health inequities.

Fridman et al. showed that while older adults, study participants with lower education levels, and those from lower-income groups may encounter obstacles like limited access to technology or unfamiliarity with digital tools, younger, better-educated, and wealthier people are more likely to adopt and benefit from EHS [22]. In a similar vein, Asiri et al. discovered that participant satisfaction with EHS differed by gender and educational level, with females reporting higher levels of satisfaction and those with less education expressing higher levels of satisfaction than those with advanced degrees [23]. These results imply that different population subgroups have different expectations for EHS and perceptions of its usability, which could affect adoption rates and satisfaction.

The digital divide presents challenges for healthcare systems aiming to implement EHS universally. The new systems significantly disadvantage populations like underserved urban areas and rural communities that have limited access to dependable internet or digital devices. Differences in digital literacy, which disproportionately impact older adults, non-native English speakers, and those with less formal education, exacerbate these disparities. Adoption of EHS may continue to be unfair and limit its ability to enhance care for underserved populations in the absence of focused interventions [32,33].

To address these disparities, healthcare systems must adopt inclusive strategies that ensure equitable access to EHS. Multi-channel communication options, for instance, which combine EHS with more conventional approaches like phone support or in-person services, can help to bridge the gap for populations that are less familiar with or inept at using digital tools. Furthermore, funding population-specific digital literacy initiatives, like workshops for senior citizens or culturally relevant instruction for non-native English speakers, can enable marginalized communities to interact with EHS more successfully [32,33].

Another critical consideration is the design and functionality of EHS platforms. Complex or poorly designed interfaces may discourage use among individuals with limited technological experience. Developing user-friendly and intuitive platforms is crucial for people with varying degrees of literacy and technological competence. EHS can be made more user-friendly for a larger range of users by including features like language support, simplified navigation, and clear, jargon-free instructions. The affordability of EHS is also impacted by equity considerations. Certain technologies, like patient portals, are frequently provided without charge, but others, like wearable technology or telehealth services that require a membership, can be prohibitively expensive. Policymakers and healthcare organizations should prioritize subsidized or free access to essential EHS tools for low-income populations, ensuring that cost does not become a prohibitive factor [34].

Addressing disparities also depends extensively on healthcare practitioners. Providers can modify their recommendations for EHS use if they are aware of and understand the obstacles that particular patient populations experience. For example, they can offer additional support to patients who are less familiar with digital health tools or direct them to resources that can enhance their confidence and skills in using EHS [35].

Finally, there is a need for more research on the social determinants of health as they relate to EHS adoption. Understanding how factors such as housing, employment, and community infrastructure intersect with digital health engagement can help identify additional barriers and inform targeted interventions. Furthermore, future studies should examine the impact of inclusive EHS strategies on reducing health inequities and improving outcomes for marginalized populations.

Strengths and Limitations

This systematic review provides a comprehensive synthesis of evidence on the impact of EHS on patient satisfaction in primary care settings. By incorporating studies from multiple regions and healthcare systems, this review captures a wide spectrum of EHS implementations, contributing to the generalizability of the findings. The review also focused exclusively on non-clinical outcomes, such as satisfaction, communication, and workflow improvements, aligning with its aim to assess workforce-driven innovations in organizational practices rather than clinical advancements. This specificity enhances the relevance of the findings to primary care management and policy. The inclusion of studies published between 2014 and 2024 ensured that recent advancements in EHS were captured, making the review particularly pertinent in the context of rapidly evolving digital healthcare technologies.

Despite its strengths, this review has several limitations. First, the methodological quality of the included studies varied, with RCTs exhibiting a "High" overall risk of bias in certain domains, such as blinding of participants and allocation concealment. These issues may limit the reliability of the findings from these trials. Despite providing valuable practical insights, observational studies are inherently susceptible to selection bias, confounding, and other methodological problems that could affect the interpretation of the results. Second, the findings were not as applicable in low- and middle-income contexts because most of the research included in the review came from high-income countries. This review did not adequately address the particular obstacles to EHS adoption that healthcare systems in resource-constrained environments face, including disparities in access, lower digital health literacy, and infrastructure limitations. Future studies from diverse socioeconomic contexts are needed to address this gap. Another limitation relates to the heterogeneity of the included studies. Variations in EHS types, patient populations, healthcare settings, and measurement methods for patient satisfaction make it challenging to draw definitive conclusions. For instance, while some studies measured satisfaction using validated tools, others relied on less standardized approaches, which could introduce measurement bias. Furthermore, although this review concentrated on patient satisfaction, it did not thoroughly evaluate associated outcomes like provider satisfaction or cost-effectiveness, which could offer a more complete insight into the effects of EHS. English restriction and not having access to Embase and other databases are among the limitations as well. Finally, depending on the published study carries the risk of publication bias, as studies that present favorable results tend to be published more frequently than those with neutral or unfavorable findings.

Implications for Practice and Policy

The findings of this review have several practical and policy conclusions. In a clinical sense, healthcare providers need to be able to employ EHS efficiently while maintaining the interpersonal aspects of care. This calls for investments in training initiatives that instruct providers in the best way to effectively incorporate EHS into their daily operations. From a policy standpoint, rules and incentives are required to promote the adoption of EHS in an equitable manner. Funding for programs that promote digital health literacy and guarantee that underprivileged communities have access to reasonably priced technology should be given top priority by policymakers.

## Conclusions

This systematic review highlights the significant potential of EHS in improving patient satisfaction in primary care. By enhancing access, communication, and efficiency, EHS can significantly contribute to the advancement of patient-centered care. Nonetheless, challenges concerning provider communication, interoperability, and health equity underscore the necessity for careful implementation and ongoing refinement of these tools. By tackling these issues and promoting inclusive, patient-centered strategies, primary healthcare systems can fully utilize EHS to provide high-quality, equitable, and satisfying care for everyone.
